# Novel *SOX2* mutation in autosomal dominant cataract-microcornea syndrome

**DOI:** 10.1186/s12886-022-02291-4

**Published:** 2022-02-11

**Authors:** Zhi-Bo Lin, Jin Li, Lu Ye, Hai-Sen Sun, A-Yong Yu, Shi-Hao Chen, Fen-Fen Li

**Affiliations:** 1 The Eye Hospital of Wenzhou Medical University, Wenzhou Medical University, Wenzhou, Zhejiang China; 2 Shanxi Eye Hospital, Xi’an People’s Hospital (Xi’an Fourth Hospital), Affiliated Guangren Hospital, School of Medicine, Xi’an Jiaotong University, Xi’an, China

**Keywords:** Congenital cataract-microcornea syndrome, Whole-exome sequencing, *SOX2*, Eye development

## Abstract

**Background:**

Congenital cataract-microcornea syndrome (CCMC) is characterized by the association of congenital cataract and microcornea without any other systemic anomaly or dysmorphism. Although several causative genes have been reported in patients with CCMC, the genetic etiology of CCMC is yet to be clearly understood.

**Purpose:**

To unravel the genetic cause of autosomal dominant family with CCMC.

**Methods:**

All patients and available family members underwent a comprehensive ophthalmologic clinical examination in the hospital by expert ophthalmologists and carried out to clinically diagnosis. All the patients were screened by whole-exome sequencing and then validated using co-segregation by Sanger sequencing.

**Results:**

Four CCMC patients from a Chinese family and five unaffected family members were enrolled in this study. Using whole-exome sequencing, a missense mutation c.295G > T (p.A99S, NM_003106.4) in the *SOX2* gene was identified and validated by segregation analysis. In addition, this missense mutation was predicted to be damaging by multiple predictive tools. Variant p.Ala99Ser was located in a conservation high mobility group (HMG)-box domain in SOX2 protein, with a potential pathogenic impact of p.Ala99Ser on protein level.

**Conclusions:**

A novel missense mutation (c.295G > T, p.Ala99Ser) in the *SOX2* gene was found in this Han Chinese family with congenital cataract and microcornea. Our study determined that mutations in *SOX2* were associated with CCMC, warranting further investigations on the pathogenesis of this disorder. This result expands the mutation spectrum of *SOX2* and provides useful information to study the molecular pathogenesis of CCMC.

## Introduction

Congenital cataract is one of the leading causes of childhood blindness [1]. The overall prevalence of congenital cataract has been reported to be 0.63 to 9.74 per 10,000 (median = 1.71) [2]. Moreover, non-syndromic congenital cataracts may occur alone or accompanied by various congenital ocular anomalies, covering from aniridia, microcornea, microphthalmia to neurological and renal diseases [3, 4]. The high genetic heterogeneity of congenital cataracts is a challenge for establishing a reliable genotype-phenotype correlation in the clinical setting [5]. Congenital cataract-microcornea syndrome (CCMC; OMIM 116200) is marked genetic heterogeneity and the association of congenital cataract and microcornea (a horizontal corneal diameter of less than 10.00 mm) without any other systemic anomaly or dysmorphism [6], appears as a distinct phenotype affecting 12-18% of heritable congenital cataract patients [7]. The molecular basis of cataract with microcornea in the absence of microphthalmia, anterior segment dysgenesis, or coloboma has not been elucidated.

Next-Generation Sequencing (NGS), also known as high-throughput sequencing, has been increasingly applied to congenital cataract [4, 8]. To date, genetic studies have identified mutations from more than 87 causative genes in non-systemic congenital cataract, and genetic mutations remain the leading cause of congenital cataracts (data from Cat-Map http://cat-map.wustl.edu/) [9]. Among these genes, mutations in at least 19 genes and loci have been reported to be responsible for congenital cataract with microcornea, including (*EPHA2, FOXE3, GJA8, CRYGD, CRYGC, MAB21L2, ATOH7, SLC16A12, BEST1, ARL2, MIP, GJA3, MAF, CRYAA, CRYBB3, CRYBB2, CRYBB1, CRYBA4, NHS*) [9].

This study identified a novel heterozygous missense mutation in the *SOX2* gene by whole-exome sequencing in an autosomal dominant CCMC family. This mutation was not observed in any of the healthy family members. With WES technologies, our study expands the spectrum and provides additional genotype-phenotype associations in CCMC.

## Materials and methods

### Family ascertainment and DNA specimens

This study was approved by the Ethical Committee of the Eye Hospital of Wenzhou Medical University and was conducted in accordance with the Declaration of Helsinki. Written informed consent was obtained from each participant. All patients and available family members underwent a comprehensive ophthalmologic clinical examination by expert ophthalmologists and carried out to clinically diagnose. Peripheral blood samples were collected from patients and available family members. A conventional DNA extraction kit (Simgen, Hangzhou, China) was used for genomic DNA extraction. In addition, blood samples from another 200 healthy controls without of eye disease or syndromes from the Han Chinese were collected.

### Whole-exome sequencing and analysis

DNA samples from affected individuals were subjected to whole-exome sequencing (WES). WES was performed using IDT’s xGen Exome Research Panel V1.0 (Integrated DNA Technologies, San Diego, USA) and Novaseq6000 platform (Illumina, San Diego, USA) for sequencing. Human reference genome (hg19/GRCh37) was used as a reference genome to align sequence reads. Protocols for next-generation sequencing and data analysis, including copy number variation analysis, have been recently published [4, 10]. Briefly, variants were filtered for rare or absent variants in the 1000 Genomes Project database (http://www.1000genomes.org/) and minor allele frequency (MAF) less than 0.01 in the Exome Aggregation Consortium (ExAC) database (http://exac.broadinstitute.org/).

### In silico analysis

In the case of missense variants, six different predictive software were applied to estimate functional effects, including SIFT (Sorting Intolerant From Tolerant) [11], MAPP (Multivariate Analysis of Protein Polymorphism) [12], MutationTaster2 [12], PolyPhen2 [13], CADD (Combined Annotation Dependent Depletion) score [12], and REVEL (Rare Exome Variant Ensemble Learner) [14]. Variants were considered if estimated to be disease-causing by at least 2 of 6 algorithms.

### Variant confirmation and segregation analysis

Sanger sequencing was used to validate variants, as well as for segregation analysis. Sanger sequencing was then performed in 200 unrelated controls for mutation validation and prevalence testing. The topological model of the SOX2 polypeptide was shown [15], and crystal structures of the wild-type and mutant proteins were predicted with Phyre2 [16] and visualized with PyMol software (Version 1.5).

## Results

### Clinical findings

**Fig. 1:**
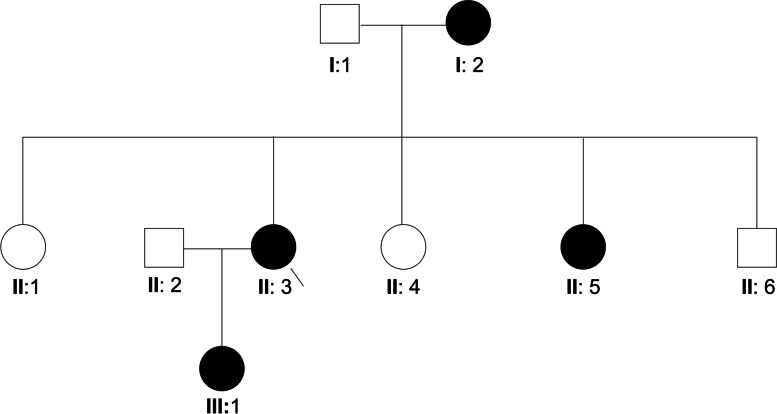
Pedigree of the congenital cataract with microcornea family. The affected family with autosomal dominant mode of inheritance. Open-square male; Open-circle female; Circle individuals with phenotype of congenital cataract with microcornea, arrowhead indicates the proband

**Fig. 2:**
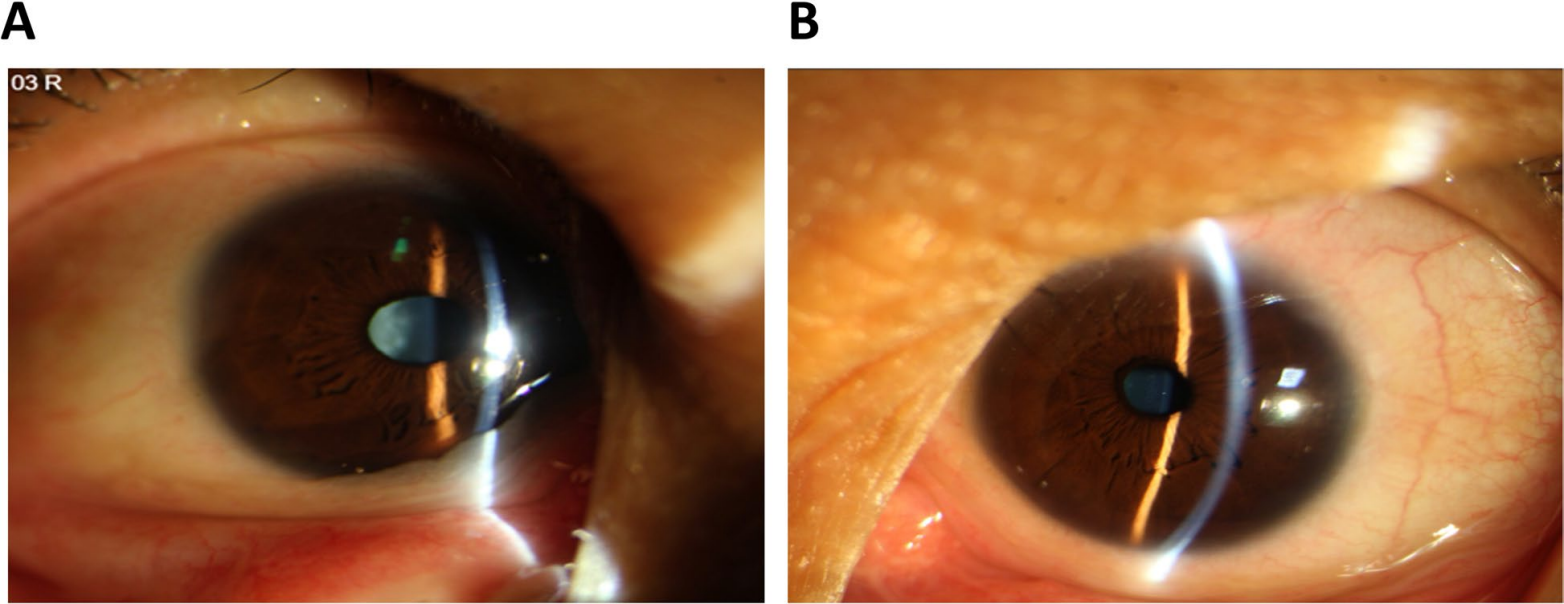
Pre-operation eye photographs and pre-operation eye photographs of the proband with congenital cataract and microcornea in a Chinese family. **A** Pre-operation eye photographs of the proband. **B** Post-operation eye photographs of the proband. She had a left eye cataract extraction in another hospital, which provided us with post-operation photos

A three-generation Chinese pedigree that consists of 9 individuals, including four affected individuals, provided the basis for the study (Fig. 1). The autosomal dominant pattern of inheritance was ascertained from the pedigree (Fig. 1). Clinical data of affected individuals are given in Table 1. The proband was a 46-year-old female who had a left eye cataract extraction in another hospital, which provided us with a post-operation photo in the left eye and a pre-operation photo in the right eye (Fig. 2). According to her medical records, the patients have congenital posterior subcapsular opacities cataract with microcornea (Fig. 2). The axial length of her right eye is 24.70 mm and 24.03 mm in the left eye; the corneal diameter is 9.5 mm in both eyes (Table 1). In addition, the proband also has nystagmus and strabismus. Her mother, sister, and daughter also have similar phenotypes with the proband. All affected individuals were reported to be affected since birth. None of the family members had any other ocular or systemic abnormalities identified after a complete physical and ophthalmologic examination.

**Table 1:** Clinical characteristic of affected patients

Patients	II:3	I:2	II:5	III:1
Relationships	Proband	Mother	Sister	Daughter
Age (Years)	46	68	37	26
Gender	Female	Female	Female	Female
UCVA(LogMAR)				
OD	0.92	1.70	1.40	0.40
OS	0.82	1.70	1.40	1.0
Axial length (OD/OS, mm)	24.70/24.03	NA	26.27/25.39	20.17/19.21
Corneal diameter (OD/OS, mm)	9.5/9.5	9.5/9.5	9.6/9.6	9.80/9.8
Bilateral/Unilateral	Bilateral	Bilateral	Bilateral	Bilateral
Microcornea (Y/N)	Y	Y	Y	Y
Corneal opacity (Y/N)	N	N	N	N
Cataract (Y/N)	Y	Y	Y	Y
Type of Cataract	Posterior subcapsular opacities	Posterior subcapsular opacities	Posterior subcapsular opacities	Posterior subcapsular opacities
Coloboma (Y/N)	N	N	N	N
Nystagmus (Y/N)	Y	Y	Y	Y
Strabismus (Y/N)	Y	N	Y	Y
Glaucoma (Y/N)	N	N	N	N
Extra ocular abnormality (Y/N)	N	N	N	N

### In silico analysis of variant in *SOX2*

After exome sequencing, data were filtered and analyzed for the causative variant of clinical phenotypes observed in patients. A missense variant, c.295G > T (p.A99S, NM_003106.4) in the *SOX2* gene, was responsible for the disease. The *SOX2* gene was located on chromosome 3 (3q26.33), encoding 317 amino acids. This variant has not been reported in genomAD, 1000 genomes databases or in the ExAC database. In predictive software, this missense variant was damaging in SIFT, MAPP, MutationTaster2, and PolyPhen-2, which showed the deleterious effect of the mutation on protein function (Table 2).

**Table 2:** In silico analysis with predictive software

	SIFT	MAPP	MutationTaster2	PolyPhen-2	CADD	REVEL
Predicted value	D	D	D	D	31	0.762

*D* Damaging, *Align GVGD* Grantham Variation/Grantham Deviation, *SIFT* Sorting Tolerant From Intolerant, *MAPP* Map Annotator and Pathway Profiler, *CADD* Combined Annotation Dependent Depletion, *REVEL* Rare Exome Variant Ensemble Learner

### Segregation analysis

**Fig. 3:**
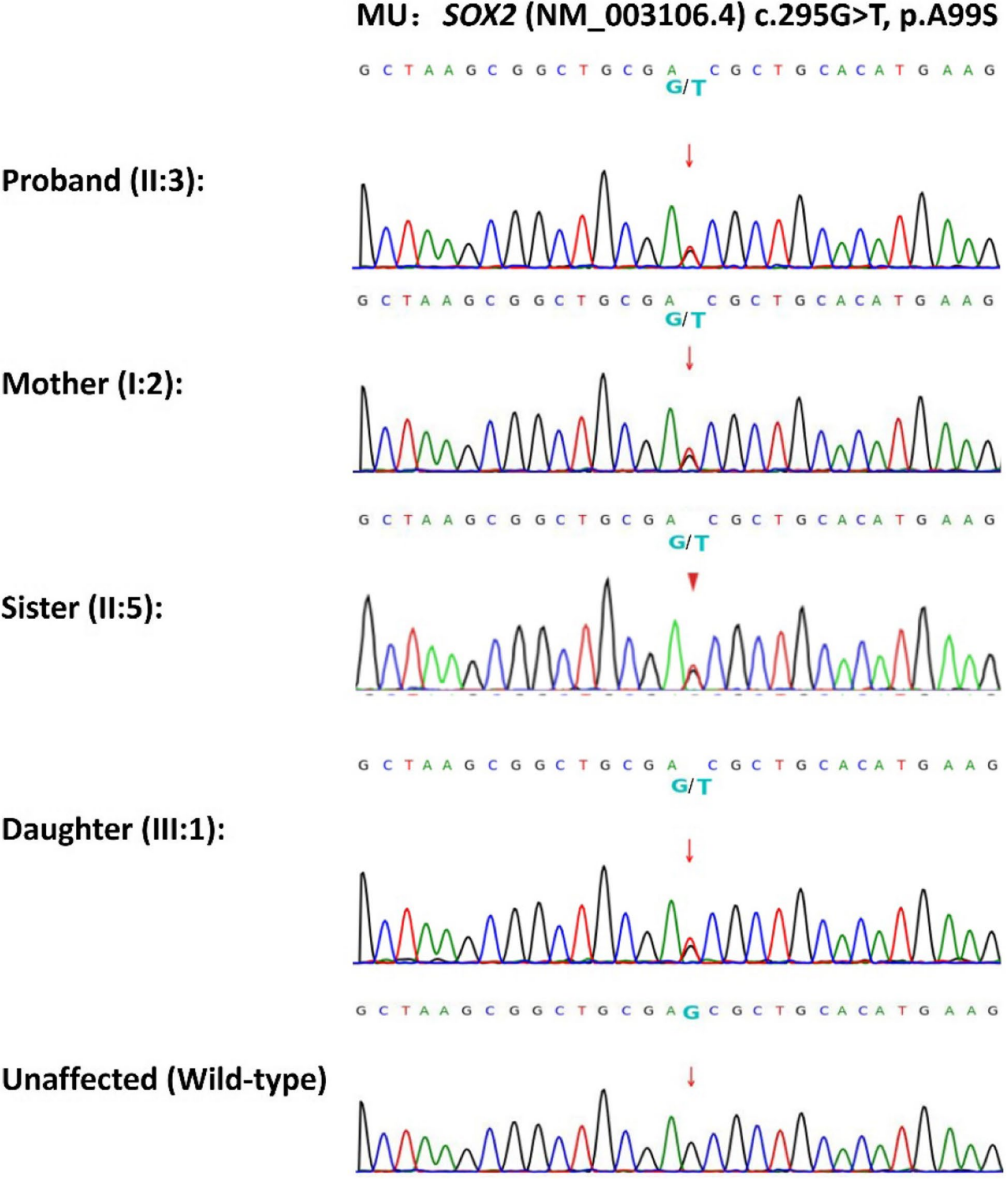
DNA sequencing profiles of the identified mutations (*upper*) and their wild-type form (*under*)

Sanger sequencing confirmed the autosomal dominant mode of inheritance of the *SOX2* gene mutation (c.295G > T, p.A99S) in the affected family. All affected family members were heterozygous for this mutation, while all unaffected were homozygous normal (Fig. 3). Besides, this variant was absent in 200 unrelated controls, suggesting it is extremely rare.

### In silico analysis

**Fig. 4:**
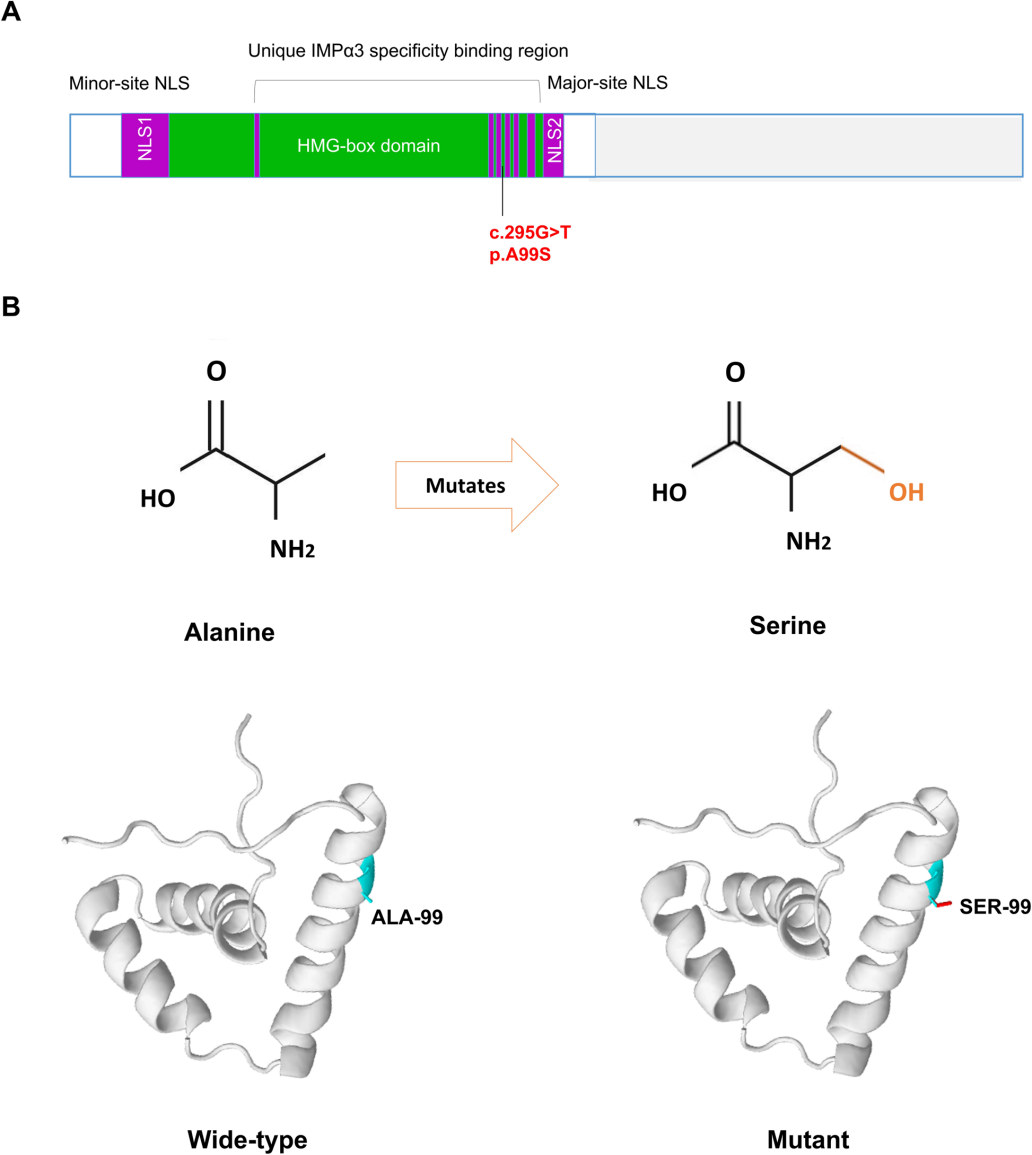
Mutations and predicted crystal structures of SOX2 protein. **A** Location of the mutations with respect to the topological model of the *SOX2* polypeptide. **B** Predicted crystal structures of wild-type (left) and mutant (right) SOX2 protein. SOX family member contains a highly conserved HMG domain (in green/purple), with NLSs positioned within the extremities of the HMG domain

We carried out comparative and structural analyses to predict the potential pathogenic impact of p.Ala99Ser on protein level. This missense mutation causes the replacement of an Alanine with a Serine in the protein product of SOX2. The topological model revealed that *SOX2* p.Ala99Ser was conserved in the high mobility group (HMG)-box domain (Fig. 4A). In addition, protein structure modeling showed that p.Ala99Ser was located in the helix and increased additional hydrogen bonds within space surrounded (Fig. 4B).

## Discussion

Our study reports the identification of a novel missense mutation in *SOX2*, c.295G > T (p. Ala99Ser), in a Chinese family with congenital cataract and microcornea. More importantly, we reveal that missense mutation in *SOX2* can cause a slight eye disorder, that is congenital cataract and microcornea, warranting further investigations on the pathogenesis of this disorder. This result expands the mutation spectrum of *SOX2* and provides useful information to the study of the molecular pathogenesis of cataract and microcornea.

Normal dosage of the *SOX2* gene are a developmentally regulated transcription factor that plays a critical role in embryonic development, particularly in the eyes and brain [17]. *SOX2* is a single exon transcription factor previously associated with anophthalmia [18, 19], microphthalmia [20], and coloboma [21]. Sox2 is involved in crystallin regulation in murine [22] and avian models [23] and humans, and *SOX2* mutations cause microphthalmia and cataracts [24, 25]. Previously, 21 of 37 patients with anophthalmia and microphthalmia carried frameshift and deletion/translocation mutations in the *SOX2* gene [26]. In terms of missense mutations, only 2 of 37 patients with microphthalmia carried missense mutations, c.131C > G and c.166C > G [26, 27], indicating that phenotype released from deletion missense mutation. Interestingly, a missense mutation in this study, *SOX2* c.295G > T p.Ala99Ser, caused a slight phenotype: microcornea and cataract.

Interestingly, this study reports that this novel mutation in *SOX2* may lead to a broader phenotypic spectrum depending on the affected partner interaction domains. In addition to this, it is possible that the genetic makeup of these partner factors, other ocular development genes, or as yet unknown factors may play a role in the phenotypic expression of *SOX2* mutations. Since expression during lens development represents an important site of SOX2 protein activity, some mutations may lead to phenotypes associated with specific loss of *SOX2*-related activity in the lens, such as cataracts and anterior segment defects. As mentioned above, the variation in phenotypes associated with, in particular, missense changes in *SOX2*, may be due to these mutations resulting in alteration of its interactions with tissue-specific protein partners rather than a complete loss-of-function [15]. The missense mutation in this study, *SOX2* c.295G > T p.Ala99Ser, is conserved in the high mobility group (HMG)-box domain [15],which is critical for correct binding to interacting proteins and to target DNA sequences [28, 29]. The phenotype of associated microcornea may be due to the induction of corneal effects by an abnormally formed lens during embryonic development and/or a reduction of *SOX2* on corneal molecular chaperones.

This study also has some limitations. Specifically, no functional analysis was performed of the identified variants in this study. Although gene function experiments and animal models are lacking, with the help of previous studies and using this as a basis, this deleterious mutation is pathogenic in combination with in silico analyses and structural comparison in *SOX2* variants.

## Conclusion

A novel missense mutation (c.295G > T, p.Ala99Ser) in the *SOX2* gene was found in this Han Chinese family with congenital cataract and microcornea. Our study demonstrated the missense mutation in *SOX2* was associated with CCMC, warranting further investigations on the pathogenesis of this disorder. This result expands the mutation spectrum of *SOX2* and provides useful information to the study of the molecular pathogenesis of cataract and microcornea.

## Data Availability

All data generated or analyzed during this study are included in this published article. And sequencing data have been submitted to NCBI SRA database: PRJNA797523.
